# b matrix errors in echo planar diffusion tensor imaging

**DOI:** 10.1120/jacmp.v2i3.2612

**Published:** 2001-09-01

**Authors:** Saïd Boujraf, Robert Luypaert, Michel Osteaux

**Affiliations:** ^1^ Biomedical MR Unit AZ‐VUB Laarbeeklaan 101, B‐1090 Brussels Belgium

**Keywords:** diffusion‐weighted magnetic resonance imaging, diffusion tensor, **b** matrix

## Abstract

Diffusion‐weighted magnetic resonance imaging (DW‐MRI) is a recognized tool for early detection of infarction of the human brain. DW‐MRI uses the signal loss associated with the random thermal motion of water molecules in the presence of magnetic field gradients to derive parameters that reflect the translational mobility of the water molecules in tissues. If diffusion‐weighted images with different values of **b** matrix are acquired during one individual investigation, it is possible to calculate apparent diffusion coefficient maps that are the elements of the diffusion tensor. The diffusion tensor elements represent the apparent diffusion coefficient of protons of water molecules in each pixel in the corresponding sample. The relation between signal intensity in the diffusion‐weighted images, diffusion tensor, and **b** matrix is derived from the Bloch equations. Our goal is to establish the magnitude of the error made in the calculation of the elements of the diffusion tensor when the imaging gradients are ignored.

PACS number(s): 87.57. –s, 87.61.–c

## I. INTRODUCTION

From the signal intensity in diffusion‐weighted images we can compute the diffusion tensor elements using Eq. (1),^1^

(1) $$\begin{array}{lll} \text{Ln}\{I(b)/I[b(0)]\} & = & -\left(\sum_{i=1}^{3}\sum_{j=1}^{3}b_{ij}D_{ij}\right)\\ & = & -(b_{xx}D_{xx}+b_{yy}D_{yy}+b_{zz}D_{zz}+2b_{xy}D_{xy}+2b_{xz}D_{xz}+2b_{yz}D_{yz}). \end{array}$$

$b_{\text{ij}}$ represents the elements of the **b** matrix of row *i* and column *j*. It contains the information on the gradient pulses^2^ ^–^ ^4^ related to the *ij* direction $(i=x,y,\;\text{and}\;z,j=x,y,\;\text{and}\;z).D_{\text{ij}}$ are the components of the symmetric 3×3 diffusion tensor **D**. *I*(**b**) is the measured signal intensity when diffusion gradients are on and *I*(0) is the measured signal intensity without gradients.

For an accurate estimation of the diffusion tensor we must take into account all diffusion and imaging gradients in the sequence as well as all their cross terms.

The purpose of this paper is to present a numerical calculation of the **b** matrix for single shot pulsed gradients diffusion‐weighted echo‐planar imaging (DW‐EPI) sequence with sinusoidal readout gradients. The contributions of the different gradients (and their interactions) to the values of the **b** matrix elements are calculated explicitly and compared.

## II. MATERIAL AND METHOD

In this paper the laboratory frame of reference with axes (*x, y*, and *z*) is the same as the image frame of reference (read, phase, and slice) direction.

Our numerical calculations were performed using Mathcad Software (Math Soft, International, UK) running on a personal computer. The **b** matrix elements in Eq. (1) were calculated using Eq.(2),^1^

(2) $$b=\gamma^{2}\int_{0}^{2\tau }[f(t)-2H(t-\tau )f(t)]{\left[f(t)-2H(t-\tau )f(t)\right]}^{T}dt,$$

where **G**(t) is the applied magnetic field gradient vector as a function of time,

(3) $$G(t)=[G_{x}(t),G_{y}(t),G_{z}(t)],$$

and its time integral

(4) $$f(t)=\int_{0}^{t}[G(t^{\prime })dt^{\prime }].$$

**H**(*t*) is the Heaviside function.

**Figure 1:**
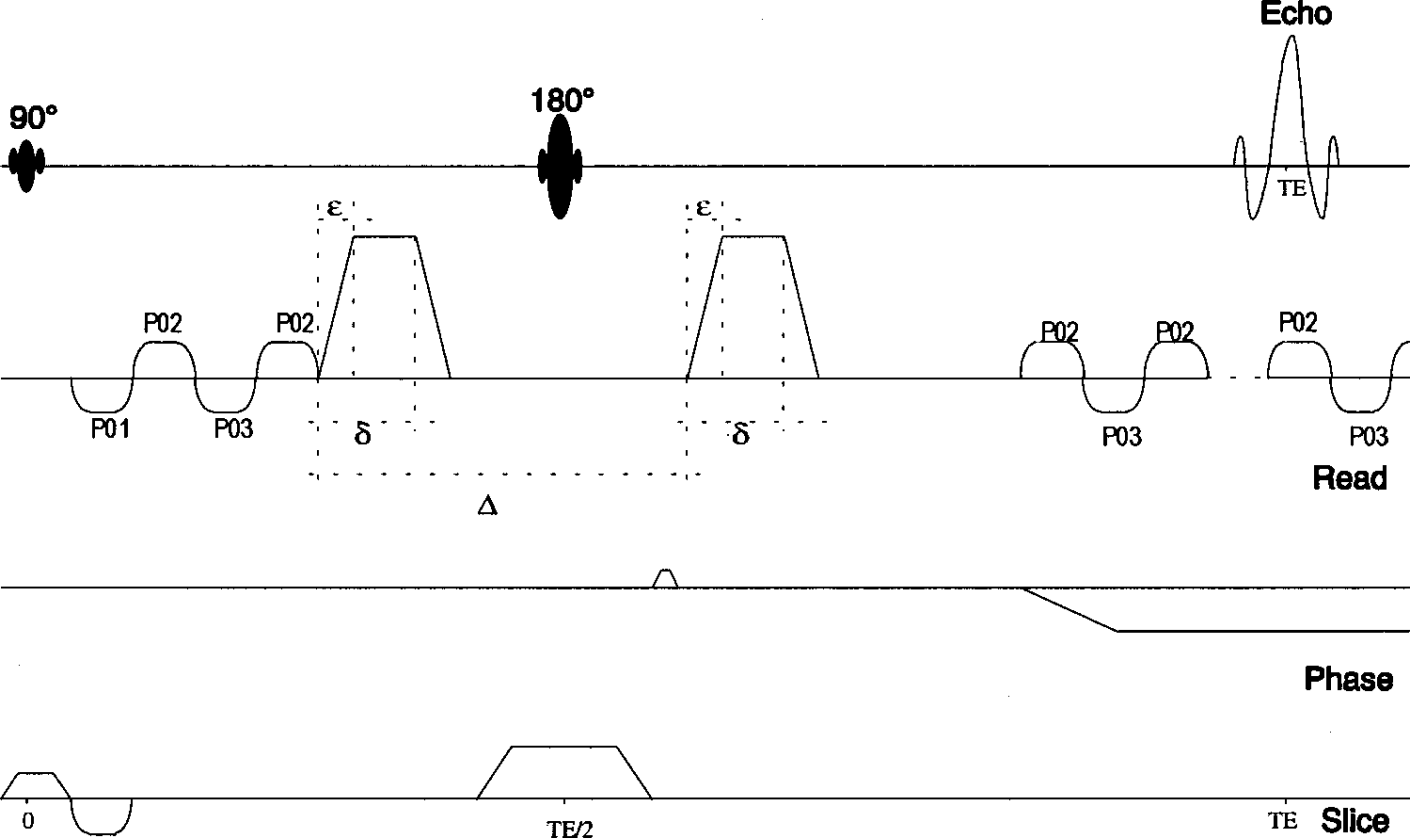
Schematic diagram of the diffusion‐weighted EPI sequence.

For our sequence (see Fig. 1), trapezoidal diffusion‐weighting pulses with a variable amplitude **G**(*t*) were applied before and after the 180° radio frequency pulse. The $b_{\text{ij}}$ elements of the **b** matrix were calculated for diffusion gradients along the phase, read, and slice directions, and along the bi‐sectors phase‐read, phase‐slice, and read‐slice. The ramp‐up and ramp‐down times of all the diffusion and imaging gradients were taken into account, including the sinusoidal readout gradients.

The full **b** factors were calculated numerically, then analytically approximated according to the Stejskal‐Tanner formula for trapezoidal pulses, including rise and fall times *ε* of the diffusion gradient pulses,^2^

(5) $$b={\left(\gamma G\right)}^{2}\left[\left(\Delta -\frac{\delta }{3}\right)\delta^{2}+\frac{\varepsilon^{3}}{30}-\frac{\varepsilon^{3}}{6}\delta \right].$$

*γ, δ*, and Δ are the gyromagnetic ratio for protons, the diffusion gradient pulse duration, and the delay between the start times of the diffusion gradients, respectively. This approximation corresponds to ignoring the imaging gradients.

The experimental validation of the different **b** matrices was performed on a 1.5 Tesla Magnetom Vision, whole body imager with a maximum gradient strength of 25 mT/m (Siemens, Erlangen, Germany), using a series of calibration measurements on an isotropic phantom (water doped with ${\text{NiSO}}_{4*}6H_{2}O$ at ~20°C). The measurement parameters were TE (echo time)=123 ms, TR (repetition time)=800 ms,  slice thickness=6 mm, FOV (field of view)=240 mm, and matrix size= 128×128. The diffusion tensor elements were calculated using both sets of values for the $b_{\text{ij}}$, and compared to results found for spin echo imaging sequence from the literature.^5^

Our magnetic resonance imager is capable of generating ±25 mT/m in 150 ms. For this sequence, the ramps of diffusion gradients were fixed to ε=700μs with a duration of δ=26 ms, and diffusion time Δ=59.7 ms.

## III. RESULTS

Tables I and II report the elements of the symmetric **b** matrix as calculated for three diffusion gradient strengths of 0, 11, and 22 mT/m. These values of diffusion gradients are the optimized ones for the use in diffusion‐weighted imaging scans in MR clinical scanners. The units of the **b** matrix elements are s/mm^2^.

**Table I:** Full b matrix elements, calculated using Eq. (2), taking into account all imaging and diffusion gradients.

	$b_{\text{xx}}$	$b_{\text{yy}}$	$b_{\text{zz}}$	$b_{\text{xy}}$	$b_{\text{xz}}$	$b_{\text{yz}}$
G(t)=(0,0,0)	0.5996	0.0339	0.5729	−0.1077	−0.5387	0.1259
G(t)=(11,0,0)	314.06	0.0339	0.5729	−0.989	−3.9612	0.1259
G(t)=(22,0,0)	1225.1	0.0339	0.5729	−1.8704	−7.3837	0.1259
G(t)=(0,11,0)	0.5996	297.04	0.5729	7.2406	−0.5387	−2.2506
G(t)=(0,22,0)	0.5996	1191.6	0.5729	14.589	−0.5387	−4.6271
G(t)=(0,0,11)	0.5996	0.0339	293.85	−0.1077	4.3409	−0.6857
G(t)=(0,0,22)	0.5996	0.0339	1184.7	−0.1077	9.2205	−1.4973
$G(t)=(11/\sqrt{2},11/\sqrt{2},0)$	160.37	148.17	0.5729	153.85	−2.9588	−1.5545
$G(t)=(22/\sqrt{2},22/\sqrt{2},0)$	618.91	595.07	0.5729	606.57	−5.3788	−3.235
$G(t)=(11/\sqrt{2},0,11/\sqrt{2})$	160.37	0.0339	146.07	−0.7309	149.87	−0.448
$G(t)=(22/\sqrt{2},0,22/\sqrt{2})$	618.91	0.0339	590.34	– 1.3541	599.05	−1.0219
$G(t)=(0,11/\sqrt{2},11/\sqrt{2})$	0.5996	148.17	146.07	5.0884	2.9117	146.16
$G(t)=(0,22\sqrt{2},22/\sqrt{2})$	0.5996	595.07	590.34	10.284	6.3621	588.78

**Table II:** b matrix elements calculated using Eq. (5), which neglects the contributions of the imaging gradients.

	$b_{\text{xx}}$	$b_{\text{yy}}$	$b_{\text{zz}}$	$b_{\text{xy}}$	$b_{\text{xz}}$	$b_{\text{yz}}$
G(t)=(0,0,0)	0	0	0	0	0	0
G(t)=(11,0,0)	298.766	0	0	0	0	0
G(t)=(22,0,0)	1195.06	0	0	0	0	0
G(t)=(0,11,0)	0	298.766	0	0	0	0
G(t)=(0,22,0)	0	1195.06	0	0	0	0
G(t)=(0,0,11)	0	0	298.766	0	0	0
G(t)=(0,0,22)	0	0	1195.06	0	0	0
$G(t)=(11/\sqrt{2},11/\sqrt{2},0)$	149.383	149.383	0	149.383	0	0
$G(t)=(22/\sqrt{2},22/\sqrt{2},0)$	597.531	597.531	0	597.531	0	0
$G(t)=(11/\sqrt{2},0,11/\sqrt{2})$	149.383	0	149.383	0	149.383	0
$G(t)=(22/\sqrt{2},0,22/\sqrt{2})$	597.531	0	597.531	0	597.531	0
$G(t)=(0,11/\sqrt{2},11/\sqrt{2})$	0	149.383	149.383	0	0	149.383
$G(t)=(0,22/\sqrt{2},22/\sqrt{2})$	0	597.531	597.531	0	0	597.531

Each line of Tables I and II represents the independent elements of one **b** matrix, corresponding to one diffusion gradient strength, in the specific direction.

Tables III and IV report the magnitude of the differences between the including and the neglecting of the imaging gradients in the **b** matrix calculation. The elements marked with three asterisks have a value less than 0.01%, which was considered insignificant for this work. The “–” corresponds to a value not compared in the appropriate table.

**Table III:** Percentage of deviation (in absolute value) between both sets of b matrix elements, corresponding to the applied diffusion gradients.

	$b_{\text{xx}}$	$b_{\text{yy}}$	$b_{\text{zz}}$	$b_{\text{xy}}$	$b_{\text{xz}}$	$b_{\text{yz}}$
G(t)=(11,0,0)	5%	‐	‐	‐	‐	‐
G(t)=(22,0,0)	2.44%	‐	‐	‐	‐	‐
G(t)=(0,11,0)	‐	1.40%	‐	‐	‐	‐
G(t)=(0,22,0)	‐	***	‐	‐	‐	‐
G(t)=(0,0,11)	‐	‐	1.64%	‐	‐	‐
G(t)=(0,0,22)	‐	‐	0.10%	‐	‐	‐
$G(t)=(11/\sqrt{2},11/\sqrt{2},0)$	7%	1%	‐	3%	‐	‐
$G(t)=(22/\sqrt{2},22/\sqrt{2},0)$	3.45%	***	‐	***	‐	‐
$G(t)=(11/\sqrt{2},0,11/\sqrt{2})$	7%	‐	2.25%	‐	***	‐
$G(t)=(22/\sqrt{2},0,22/\sqrt{2})$	3.45%	‐	1%	‐	***	‐
$G(t)=(0,11/\sqrt{2},11/\sqrt{2})$	‐	1%	2.25%	‐	‐	2.10%
$G(t)=(0,22/\sqrt{2},22/\sqrt{2})$	‐	***	1.17%	‐	‐	1.50%

**Table IV:** Percentage of deviations between b matrix elements corresponding to directions that are off diffusion gradients. The comparison involves b matrix elements corresponding to non‐null diffusion gradients and b matrix elements corresponding to null diffusion gradients. This result shows the contribution of the interaction of diffusion and imaging gradients to the elements corresponding to directions that are off diffusion gradients.

	$b_{\text{xx}}$	$b_{\text{yy}}$	$b_{\text{zz}}$	$b_{\text{xy}}$	$b_{\text{xz}}$	$b_{\text{yz}}$
G(t)=(11,0,0)	‐	***	0.20%	***	1.32%	***
G(t)=(22,0,0)	‐	***	***	***	***	***
G(t)=(0,11,0)	0.20%	‐	0.20%	2.40%	0.20%	0.75%
G(t)=(0,22,0)	***	‐	1.20%	***	***	***
G(t)=(0,0,11)	0.20%	***	‐	***	1.40%	0.23%
G(t)=(0,0,22)	***	***	‐	***	***	***
$G(t)=(11/\sqrt{2},11/\sqrt{2},0)$	‐	‐	2.25%	‐	2%	1%
$G(t)=(22/\sqrt{2},22/\sqrt{2},0)$	‐	‐	3.45%	‐	***	0.50%
$G(t)=(11/\sqrt{2},0,11/\sqrt{2})$	‐	***	‐	***	‐	***
$G(t)=(22/\sqrt{2},0,22/\sqrt{2})$	‐	0.02%	‐	***	‐	***
$G(t)=(0,11/\sqrt{2},11/\sqrt{2})$	0.01%	‐	‐	3.40%	2%	‐
$G(t)=(0,22/\sqrt{2},22/\sqrt{2})$	***	‐	‐	1.72%	1.06%	‐

For validating the **b**‐matrix results and for showing their effects on the degree of accuracy obtained for the diffusion tensor elements, these were calculated using quantitative diffusion analysis.^6^ ^,^ ^7^ For $D_{f}$, the diffusion tensor calculated using **b** matrices, taking into account all imaging and diffusion gradients, and $D_{s}$, the diffusion tensor calculated using the **b** matrices in which the imaging gradients are neglected, we found (in mm^2^/s)

$$D_{f}=\left(\begin{array}{lll} 2.08\times 10^{-03} & 4.33\times 10^{-06} & 1.56\times 10^{-05}\\ 4.33\times 10^{-06} & 2.02\times 10^{-03} & 5.38\times 10^{-06}\\ 1.56\times 10^{-05} & 5.38\times 10^{-06} & 2.13\times 10^{-03} \end{array}\right),$$

and

$$D_{s}=\left(\begin{array}{lll} 2.27\times 10^{-03} & 1.84\times 10^{-05} & 2.39\times 10^{-05}\\ 1.84\times 10^{-05} & 2.14\times 10^{-03} & 1.36\times 10^{-05}\\ 2.39\times 10^{-05} & 1.36\times 10^{-05} & 2.18\times 10^{-03} \end{array}\right)$$

These results were evaluated by comparison with the data for the diffusion coefficient of water,^5^ ${D_{o}=(1.96\pm 0.5)}^{*}10^{-03}{\text{mm}}^{2}/s$ as shown in Table V.

**Table V:** Relative deviation of trace/3 of $D_{f}$ and $D_{s}$ from literature data for the diffusion coefficient of water.^5^

	$D_{\text{xx}}$	$D_{\text{yy}}$	$D_{\text{zz}}$
$(D_{f}-D_{o})/D_{o}$	(5.77±0.03)%	(2.97±0.07)%	(7.98±0.02)%
$(D_{f}-D_{o})/D_{o}$	(13.66±0.04)%	(8.41±0.03)%	(10.10±0.03)%

## IV. DISCUSSION AND CONCLUSIONS

For the approximated **b** matrix elements, it is expected that off‐diagonal elements of the diffusion tensor will be significantly different from zero, and the diagonal elements of the diffusion tensor will be significantly different from each other, even for an isotropic phantom.

This is because a given percentage error in the **b** matrix element produces the same percentage error in the corresponding element of the (statistically) estimated diffusion tensor element, but of an opposite sign.^8^ An underestimated **b** matrix element should yield an overestimation in the corresponding diffusion tensor element, and vice versa.

Therefore, errors in **b** should cause errors in estimation of diffusion tensor **D**, which should make the estimated **D** for isotropic phantom (water) deviate from isotropy.

The off‐diagonal elements of $D_{f}$ and $D_{s}$ are negligible, as they are smaller than the error associated with $D_{o}$ measurements.

In comparison to $D_{o}$, the errors made in the diagonal elements of $D_{s}$ vary from about 8% to 14% according to the importance of imaging gradients neglected in the corresponding diffusion direction. While for the diagonal elements of $D_{f}$ the errors are lower, varying from about 3% to 8%.

Other potential sources of errors in the calculated diffusion tensor elements can be attributed to:

i) The eddy‐currents induced in the magnetic resonance imager, resulting from fast gradients switching. In the DW‐EPI sequences, the eddy‐currents depend on the magnitudes and the directions of the diffusion gradients. This explains the nonuniformity of the errors made in the diagonal elements of the diffusion tensor.
ii) The fast gradient switching induces a Lorentz force on the gradient coil set which in turn causes a mechanical motion of the coils and the resulting vibration. The phantom would not be likely to vibrate if it was not in an MR scanner. The fast gradient switching simply generates higher vibrational frequencies at larger amplitudes than would be present in a conventional MR scanner.
iii) Noise resulting from the process of deriving the diffusion tensor elements from the diffusion‐weighted echo‐planar images.

As expected the error is higher when using weak diffusion gradients. The effect of this systematic error on the estimation of the diffusion tensor elements for an isotropic phantom with known diffusion coefficients:

‐ The diffusion tensor is, to a good approximation, diagonal, as expected for an isotropic medium.

‐ In each case, we obtain diagonal elements that overestimate the true value of the diffusion coefficient.

‐ This overestimation is up to three times smaller when imaging gradients are not neglected.

Experimental errors could be generated by noise accumulated during the measurement of the diffusion‐weighted images. Further investigation about the effects of experimental error on the accuracy of diffusion tensor components is needed.
